# Social prescribing of cultural opportunities to support health and wellbeing: the importance of language, community engagement, and inclusion in developing local approaches

**DOI:** 10.1186/s12875-025-02835-9

**Published:** 2025-05-08

**Authors:** Anna Dadswell, Hilary Bungay

**Affiliations:** 1 School of Allied Health and Social Care, Faculty of Health, Medicine and Social Care, Anglia Ruskin University, Bishop Hall Lane, Chelmsford, CM1 1SQ UK; 2 School of Allied Health and Social Care, Faculty of Health, Medicine and Social Care, Anglia Ruskin University, East Road, Cambridge, CB1 1PT UK

**Keywords:** Social prescribing, Culture and health, Social prescribing link workers, Healthcare professionals and community connectors

## Abstract

**Background:**

There is growing evidence for the role of culture in supporting health and wellbeing, including as part of social prescribing provision. This study set out to explore the existing cultural provision and mechanisms for connecting people to cultural opportunities as part of a local social prescribing offer in the UK and how it could it be strengthened to better support health and wellbeing. A broad view of culture was adopted encompassing the creative and digital industries, heritage, food, hospitality, nature, greenspaces, and sport. It covers activity associated with the artforms and organisations such as collections, combined arts, dance, libraries, literature, museums, music, theatre and the visual arts.

**Methods:**

A qualitative exploratory descriptive approach using focus groups was employed to explore the perspectives and experiences of social prescribing and the cultural provision in an area of the East of England. Six focus groups were conducted with social prescribers, community connectors, healthcare professionals, cultural providers, adults with lived experience of adverse health, and young people. Data was analysed using a reflexive thematic approach.

**Results:**

Findings from the research highlight the need for a clear and shared understanding of culture and health and the link to social prescribing. Barriers for connecting people with culture and health opportunities in the area were identified including gaps in provision and processes, challenges due to language and terminology, accessibility issues for marginalised groups, and issues around funding for transport and sustainable and equitable provision of cultural opportunities.

**Conclusions:**

This study indicated that to engage local communities in social prescribing and the development of cultural provision for social prescribing, proactive outreach community strategies are required. This could be achieved by involving community leaders, organisers, connectors, and representatives. In addition to promote the concept of culture for health and social prescribing and engage the wider community it was suggested that community members should be involved in and contribute to culture and health social prescribing locally through volunteering, roles for students, training opportunities, and befriending or buddy schemes.

## Introduction

In this study concepts of culture and cultural opportunities were adopted encompassing the creative and digital industries, heritage, food, hospitality, nature, greenspaces, and sport, and covering activity associated with the artforms and organisations such as collections, combined arts, dance, libraries, literature, museums, music, theatre and the visual arts. There is a growing body of multidisciplinary research demonstrating the positive impacts of engaging with cultural opportunities. This includes the arts, music, creativity, heritage and nature, exercise and physical activity, and social or philosophical activities – on people’s health and wellbeing [1–7]. Such impacts include increased social interaction and decreased loneliness; embracing healthier lifestyles and physical activity; reduced anxiety and depression; increased confidence to manage own health, a reduction in health service usage; general improved wellbeing; enhanced social cohesion, employment and skill development, and economic growth [3–6, 8–10]. However, culture and health are generally dealt with in distinct sectors of our society and the pathways for connecting people with cultural opportunities to support their health and wellbeing are often lacking. Social prescribing is a mechanism for addressing this disconnect, aiming to promote health and wellbeing through non-clinical, community-based approaches. It also supports the United Nations Sustainable Development Goal (SDG 3) “To ensure healthy lives and promote well-being for all at all ages” [11].

Social prescribing has been gaining momentum around the world [12, 13]. Recent research sought to establish internationally accepted conceptual and operational definitions of social prescribing using a multidisciplinary panel of experts [13]. Consensus was reached on the following short definition (alongside a more comprehensive long definition and operational definitions):

*“Social prescribing is a means for trusted individuals in clinical and community settings to identify that a person has non-medical*,* health-related social needs and to subsequently connect them to non-clinical supports and services within the community by co-producing a social prescription – a non-medical prescription*,* to improve health and well-being and to strengthen community connections.”* [13: p.9)

While this definition brings shared structure and meaning to the concept, the authors emphasise how it can account for nuances around the world. It also allows for greater flexibility and expansion of how it may be understood within local community approaches, particularly in terms of social prescribing taking place in both clinical and community settings.

In the UK, social prescribing is a key component of Universal Personalised Care and has been incorporated into the National Health Service (NHS) in England with significant investment in social prescribing link workers [14–18] and the launch of the National Academy for Social Prescribing (NASP) in 2019. Meanwhile, other initiatives and policy have encouraged the integration of culture and health that include or align with social prescribing. For example, the recent Creative Health Review provides examples of the potential of creative health to help tackle issues in health and social care [19]. In addition, the Culture, Health & Wellbeing Alliance advocates for creativity and cultural engagement to transform our health and wellbeing. Social prescribing has been described as an innovative form of integrated care [20] and the establishment of Integrated Care Systems (ICSs) in 2022 in England intended to build and strengthen partnerships between the NHS, councils, voluntary sector, and others to “improve health and care services – with a focus on prevention, better outcomes and reducing health inequalities” [21: np). Approaches to population health have traditionally been based on a deficit model which focuses on issues such as deprivation, illness and lifestyle behaviours which damage health. The emphasis on such needs and priorities have tended to overlook the role that individuals and communities can have in promoting and maintaining health [22]. ICSs support community assets and Asset Based Community Development (ABCD) representing a shift in a social deficit approach to one where community assets are identified and supported [23].

Social prescribing aligns with the mobilisation of community assets agenda, which seeks to identify existing assets, skills and resources within communities to promote wellbeing and resilience [24]. Asset-based community development approaches are gaining increasing traction in UK public health policy making [23, 25]. Asset based approaches and indeed social prescribing itself – can be seen as working towards greater integration of primary care and the community. Community-Enhanced Social Prescribing (CESP) has been advocated for to take account of the capacity of communities to meet individual need, with the suggestion that those in social prescribing roles should act as a *“catalyst in helping to build community*,* as well as individual*,* capacity”* and *“formulate a bridge between the two”* [26: p.184). However, to achieve this there is a need to understand ‘community’ and the meaning an individual attaches to the communities they identify with. Morris et al. envisaged a theory of change for CESP outlining the processes whereby the assets, networks, and resources embedded in local communities are brought together to create an enabling environment that supports people in improving their wellbeing [26]. Essentially, for CESP this entails a form of asset-based community development to identify and build close partnerships with voluntary, community and social enterprise (VCSE) sectors to identify local cultural and community opportunities and engage with local communities.

In this article, we reflect on a localised research study that explored the social prescribing of cultural opportunities to promote health in an area of the East of England. This represented the initial step in a larger programme of work to improve health and wellbeing across the community through engagement with cultural experiences. The aim of the research was to determine what the existing social prescribing for culture and health across Chelmsford looks like and how it could be strengthened. The findings from the research will be directed into recommendations for developing approaches to the social prescribing of cultural opportunities to promote health in local, national and international settings.

## Methods

### Background context

In March 2023, a local charity Culture Chelmsford published the first district-wide cultural strategy with the ambition to see the district *“celebrated as a culturally ambitious place that connects our innovative heritage of science and engineering with a vibrant future of wellbeing*,* environment and creativity*,* transforming our peoples’ lives”*. The Strategy has three themes, one of which is to improve health and wellbeing with the goal that: *“by 2033 there will be a measurable improvement in mental and physical health and the overall wellbeing of our people as a consequence of participating in cultural experiences”.* As the first step in understanding culture and health in the local population the charity established a Culture & Health Working Group (CHWG). This was a group of stakeholders and included representatives from across the NHS and healthcare providers, community and charity organisations, leisure and cultural organisations, education institutions, and individuals with expertise in fields of culture, health, or their intersection. The partnerships and discussions facilitated through the CHWG led to the initiation of this exploratory research to address the research question: What does existing social prescribing for culture and health across Chelmsford look like and how could it be strengthened? Researchers from Anglia Ruskin University worked closely with the charity, the CHWG, and the local voluntary service Chelmsford Council for Voluntary Service and with their local social prescribing service to explore the existing mechanisms for connecting people with culture and health opportunities, what is/is not working well, and where the gaps are. To answer the research questions an exploratory qualitative approach was selected and the research design comprised three stages:

1. A workshop with the CHWG to establish the context for culture and health social prescribing in the local area which includes a city and its surrounding district with a total population of around 181,500 [27].
2. Focus groups with six key stakeholder groups (focus group members are referred to as participants throughout) to explore culture and health social prescribing in the local area in greater depth and identify key issues.
3. Reflexive thematic analysis including meeting with the CHWG to reflect on the findings and develop recommendations for strengthening culture and health social prescribing in the local area.

Ethical approval was granted by the Anglia Ruskin University Faculty of Health, Medicine and Social Care Research Ethics Committee and all participants gave informed consent before contributing to the research.

### Data collection

The extensive knowledge, expertise, and networks of the CHWG was utilised to establish the current local context for connecting people to culture and health opportunities. A workshop with CHWG members (stakeholder group) was conducted online in February 2024 using an open board Miro© [28]. A Miro is an on-line collaborative whiteboard platform which allows the sharing of thoughts and ideas using digital sticky notes, it can be used in the same way as non-digital ‘sticky notes’ as a data collection method [29]. The Miro enabled the group members to respond collectively to three key questions:

1. What culture and health opportunities are you aware of in the local area?
2. How are people currently connected into these opportunities and what are the barriers to access?
3. What gaps do you see in the current culture and health social prescribing provision?

CHWG members were also able to add to the Miro Board individually for two weeks after the workshop. A total of 14 CHWG members contributed their responses to the questions and all contributions were collated and sorted. Data related to the first question of the CHWG workshop is specific to the local context and therefore not included here.

The data gathered from the CHWG workshop informed subsequent focus groups in April and May 2024 with research participants from six key local groups: cultural providers, health practitioners, social prescribers, community connectors, adults with lived experience of adverse health, and young people. It is important to note at this point that social prescribing link workers may have alternative titles in different locations including ‘community connectors’ and ‘care navigators’ but who essentially perform the same role [30].

### Recruitment

Participant recruitment was facilitated through the Culture & Health Working Group (CHWG). A short summary of the research and recruitment criteria was shared via email to relevant networks via the Culture & Health Working Group (CHWG) who were partners in this research. Neither the researcher nor members of the CHWG approached individuals directly. Prospective participants who saw the summary and wanted to take part contacted the research team by email, who then sent the participant information sheet and consent form by email. If they decided to take part, they returned the signed consent form. At this point a doodle poll was sent to those who returned the signed consent form to enable a date and time to be set for the online focus group. Individuals could decide to change their mind and withdraw from the research at any time.

The focus group questions covered the following topics:

- Perspectives and understandings around the connection between culture and health as well as what is meant by social prescribing.
- Current provision of cultural opportunities that could support health and wellbeing locally.
- Current pathways to connect people to cultural opportunities, both formal referrals through healthcare systems and informal connections through community networks.
- Examples of where social prescribing to cultural opportunities has worked well or not worked so well.
- What is missing from the current provision and pathways and what they would like to see - how social prescribing could be strengthened to better connect people with cultural opportunities to support their health and wellbeing.

A total of 36 people took part across the focus groups, with further details in Table 1. Adults and young people who joined the in person focus groups received £20 vouchers as a thank you. These participants represented a diversity of backgrounds, though demographics were not recorded due to the small numbers who took part and considerations around anonymity. Focus group discussions lasted between 40 min and 1 h 13 min and were audio-recorded and professionally transcribed.

**Table 1:** Focus group details

Key group	Description	Format	Number of participants
Cultural providers	Representatives from various cultural organisations/venues, including some specifically in outreach/engagement roles, recruited mostly through the Local Cultural Education Partnership.	Online	7
Health practitioners	Health practitioners in various roles mostly working in the NHS, including Care Coordinator and Health & Wellbeing Coach.	Online	5
Social Prescribers	Social prescribing link workers and those in similar roles, recruited through the local Chelmsford Council for Voluntary Service Social Prescribing Team and Network.	Online	7
Community connectors	Community leaders and organisers who have a role in connecting people with cultural opportunities, mostly working in the voluntary sector.	Online	8
Community members with experience of adverse health	Adult community members based locally with lived experience of adverse health and were all also working or volunteering in the cultural and/or voluntary sector.	In person	4
Young people	Young people aged 16–25 based in the city who were part of the Local Cultural Education Partnership Youth Forum.	In person	5

### Data analysis

Inductive reflexive thematic analysis guided by Braun and Clarke [31] was conducted on the data related to the second and third question of the CHWG workshop and the focus group transcriptions. The analysis took a semantic critical realist approach whereby the analysis was data led and followed a semantic approach looking at surface level meaning of the data. Coding and theme development reflect the explicit content of the data based on participants beliefs and assumptions to help understand the qualitative perspectives, experiences, and reflections of participants. The process involved familiarisation and coding the data, generating initial themes, developing and reviewing then refining the themes, and finally writing up. This process was conducted predominantly by the first author with support from the second author throughout and particularly in refining and writing up the themes. The findings were presented to the CHWG who collectively discussed the implications in terms of recommendations from the research and the possibilities for informing potential pilot projects for strengthening culture and health social prescribing locally. Recommendations were subsequently written by the researchers and reviewed and agreed by all partners. The findings presented here focus specifically on three critical issues that were identified in the data: terminology, engagement, and inclusion. All of which are important for social prescribing services more widely across the UK but also should be considered in other countries where social prescribing has been or is in the process of being implemented.

### Findings

Three main themes were identified in the data, language and understandings of social prescribing, and understanding of social prescribing, community engagement in development and delivery, and inclusion of marginalised individuals and communities all of which are central to the initial step in developing the wider approach, as well developing collaboration between healthcare providers and community organisations and the voluntary sector.

### Language and Understandings of social prescribing

The language of social prescribing was a key issue across the findings, resulting in different understandings and perceptions across individuals and groups that may compromise how social prescribing approaches are delivered and received. When asked what is meant by social prescribing, those in the social prescribers focus group described the formal process through health services that they were involved in. In line with recent definitions referred to above [13] they talked about a person-centred approach that involves building a relationship with the person they are supporting and finding out what matters to them, before exploring opportunities and supporting them to engage with their preferred activity.

*[W]hen we meet the patients*,* usually we ask them what matters to them the most… asking more questions and listening… Then*,* for example*,* if they love gardening we’ll refer them to the garden project and then that connects them with lots of different age groups and arts and crafts….* (Social Prescribers Focus Group)

Importantly, they also emphasised the wider factors that may be stopping that person from engaging, such as social isolation, mental health concerns, or their financial situation, and the significant initial work that is often needed over an extended period before they can begin to engage with a cultural activity.

*…not everybody’s ready to take that step into leaving the home or actually going somewhere on their own… There are always other barriers… the financial situation… we kind of have to tackle [those] things first*,* before we can actually get them to the groups and clubs.* (Social Prescribers Focus Group)

#### The role of social prescribers

For participants in other focus groups, some had heard of the term social prescribing but were unaware that a formal social prescribing role existed, while others – including some healthcare practitioners – did not know the extent of what social prescribers do.

*I think there is a lack of understanding amongst the population in terms of what social prescribers actually do… I mean there is a lack of clarity even between professionals*,* let alone the service users*,* so I think there needs to be education on both sides.* (Health Practitioners Focus Group)

Despite this, understandings of social prescribing were evident in the examples of good practice identified across the health practitioners, cultural providers, and community connectors focus groups.

*We’ve got some really good social prescribers*,* in our area*,* that are just brilliant. They just seem to have an abundance of*,* a huge directory of*,* community activities*,* groups they can join*,* support networks and so on. It’s an invaluable service because it’s a non-clinical service that is provided by the NHS… who can help with the mental health and the wellbeing of patients*,* we don’t necessarily want to always prescribe medication. It might just be that they need someone to point them*,* to signpost them really*,* to some areas and aspects in their life that might benefit them and to signpost people to ways of achieving goals with local community events and so on.* (Health Practitioners Focus Group)

Some participants viewed social prescribing as a valuable alternative to traditional healthcare providing access to support beyond medical treatment; though young people emphasised that this should complement rather than replace medical options.

#### Defining the boundaries of social prescribing

While there was a general understanding of social prescribing as signposting people to social and cultural opportunities, it was difficult to define boundaries around this in the community context. For example, one participant who was an arts practitioner questioned if because they were recommending people to attend cultural venues or engage with cultural opportunities whether that made them a ‘social prescriber’ or whether it was a role specific to the NHS. There were other examples throughout the findings of how people are connected to cultural activities through community leaders and organisers. One participant highlighted how important it was to have this pathway outside of formal referrals to social prescribers through the NHS given the various barriers to accessing healthcare particularly for marginalised groups. Additionally, concerns were raised about potential negative perceptions of the term social prescribing and whether there was a need for it – particularly in relation to the more informal mechanisms within the community for bringing people together and connecting people with social and cultural opportunities. One participant felt the term was alienating and stated:

*We do it*,* but I’ve never called it social prescribing before.* (Community Connectors Focus Group)

This echoed the CHWG workshop, where the branding of social prescribing was identified as a key issue. Some felt a more distinctive and easily understandable term is needed, while others suggested a campaign to raise awareness around social prescribing and the positive impacts of engaging with cultural opportunities on health and wellbeing would be beneficial.

### Inclusion of marginalised individuals and communities

Members of the CHWG identified barriers around language for those who do not speak English. Additionally, some people may be unable to read and understand written words, presenting challenges for pathways that utilise written advertisement online or in print. This emphasises the need for inclusive communication of opportunities and activities that are appropriate for diverse audiences.

In the health practitioners focus group concerns were also raised around the health inequalities that may affect certain cultures and create cultural barriers to accessing healthcare – such as discrimination, language barriers, and where people live, all of which need to be challenged and broken down. Furthermore, physical limitations, disabilities, and mental health challenges all represent barriers to engaging with culture and health opportunities. People facing these challenges may need support in accessing and participating in activities, and without the support infrastructure they are often reliant on personal support networks.

*Certain cultures tend to live out of the city centres so… there’re quite a lot of health inequalities*,* it’s harder for certain people to use public transport*,* to even attain driving licences and so on*,* to access any resources that are out there really*,* whether it be health-wise*,* wellbeing*,* or even on a social level*,* any clubs*,* community groups*,* and so on can be quite hard depending on what culture you are from.* (Health Practitioners Focus Group)

#### Inclusion of marginalised groups

Across the focus groups, but particularly in the community members focus group, participants raised concerns around the inclusion of various marginalised groups such as refugees and asylum seekers, people from Black, Asian, and Minority Ethnic (BAME) groups, people with physical disabilities or special educational needs and disabilities (SEND), people experiencing poor mental health, older people who may be frail, and people experiencing social isolation and loneliness. Some of the issues these people might face include feeling judged by others, fear and not feeling safe, not feeling represented, a lack of confidence, language barriers, accessibility issues, and needing specific support to access or engage with cultural activities.

*So what we need to do is make more people feel at home and able to express themselves*,* in their culture*,* in their language*,* activities. Someone said*,* compassion*,* be patient with them. And give them opportunities*,* like volunteering*,* all sorts of roles in society*,* then they feel they belong… in [Name removed for confidentiality]*,* when they come the first day*,* volunteering… different learning disabilities or different backgrounds. The first day*,* they’re very closed and no confidence. But when they start doing stuff*,* they gain confidence*,* they start smiling*,* they start talking. It’s an amazing difference.* (Community Members Focus Group)

In the CHWG workshop an important point was raised in relation to carers and the need to offer support and respite opportunities to carers who are key to facilitating the participation of those with physical and mental health needs.

Young people talked about the importance of representation and being able to see yourself and people like you included in cultural activities and events. One participant in the community members focus group shared how having a severe health condition and a son with SEND meant that she needed someone to come with her to access events in the park, for example. Meanwhile, a participant from the social prescribers focus group pointed out that they would be unlikely to be working with people who could go out and access social and cultural opportunities on their own; and the people that they see need a relational approach to work together to overcome various barriers.

*I think there’s something broader… thinking about what could prevent people from getting involved in things*,* particularly things like confidence… and finding ways… to help them to access them. To make that first step… particularly after COVID*,* people are feeling less confident about joining groups… how can some of those more practical matters be addressed to make sure that where there is provision people feel that it’s for them*,* that they can go to it*,* that they feel confident to go to it and can become part of that activity?* (Community Connectors Focus Group)

#### Support needs

Specific support was identified as needed to address the concerns around inclusion, and instances were provided in the focus groups. For example, one participant suggested a paid position of ‘Outreach Officer’ who was there to support people in accessing cultural opportunities. Other examples included outreach and support from community leaders and representatives, particularly for refugees and asylum seekers; befriending or buddy schemes encouraging volunteering and providing opportunities for students; providing language support and cultural activities to support language learning; clear and detailed information about accessibility and someone who can be contacted to discuss any specific support required.

*Instead of signposting them*,* getting them to access it themselves*,* physically taking them*,* accessing with them. Supporting them in accessing. And then wean them into it. Outreach volunteer work…* (Community Members Focus Group).

#### Financial implications

Moreover, it was recognised that many within these groups face additional challenges around the cost of engaging in cultural opportunities, including the cost of travel and the time it takes to attend. However, it was also noted that given the cost-of-living crisis it was not just those in marginalised or vulnerable groups who faced financial challenges to engaging in cultural activities.

*Theatre*,* again*,* it’s really expensive*,* very exclusive… who can afford to go to the theatre? Certainly not a lot of people with mental health diagnoses*,* who are on benefits or whatever. They’re very often due to obviously being on restricted incomes*,* they can’t afford to access- They can’t afford to go to the cinema… They can’t afford to go and eat out… So people who are on a low income generally*,* due to whatever circumstance*,* are very excluded from cultural life*,* because there’s not really much that they can do for free.* (Community Members Focus Group)

The lack of subsidised culture and health schemes in the area – aside from those provided through the Recovery College (an education based centre providing information, networking and skill development for managing mental health, wellbeing and daily living [32]), the Active Health Scheme (where health professionals refer individuals to the local gym) and a community space for visual arts activities provided by a third sector organisation was identified as an issue. Participants highlighted the need for comprehensive funding to provide more such schemes, these schemes provide additional benefits as there is often integrated support, with someone to meet individuals at reception and look after them while taking part in the activity; social prescribers reported that they were more comfortable referring people to schemes with such support.

### Community engagement in development and delivery

It was emphasised that the development of cultural provision for health requires community engagement. Health practitioners and young people particularly discussed this focussing on overcoming the barriers to accessing cultural activities and ensuring that provision is inclusive of diverse communities to help address the current low uptake of some cultural activities.

*[I]f you’re offering something that’s not within their culture or what they like to do… that’s not going to be worthwhile… it’s more about learning… getting to know your communities and what kinds of things they like. Then*,* actually*,* you can be more proactive at providing stuff within the community which kind of fits with people… I do see a lot of programmes… where they’re really great on paper… then they have a really bad or poor uptake… They need to do more research.* (Health Practitioners Focus Group)

However, it was also highlighted, by members of the health practitioners focus group that it was challenging to get people to contribute to their Patient Participation Group and that there was generally a low uptake by the community of the opportunities to be consulted on local health issues. This indicates the need for careful consideration about how to effectively engage communities and find out what they want and ensure representation from all parts of the local community.

#### Supporting engagement

All the focus groups suggested community outreach to promote cultural opportunities and support people to engage with them. Those in the young people focus group also suggested that cultural providers could promote the opportunities for young people through schools, they felt this may also create more demand for non-traditional sports or careers in the creative sector. Similarly, the community members focus group participants highlighted the need for community leaders or representatives to bring cultural opportunities to their communities and provide support in understanding the opportunity and having confidence to get involved.

*…you need people to liaise with the communities… support people and encourage them to come*,* give them the confidence to come. Maybe give them a bit of support when they’re there*,* if they need help with language… [or] confidence. Because very often*,* maybe people – refugees – will see an event going on in the pub*,* but then because they haven’t heard about it*,* they don’t know what it is. They might want to approach*,* they might be interested to know what it’s about*,* but then they feel a lack of confidence in approaching*,* and people might judge them… They don’t know whether they’re going to be welcomed there or whether they’re going to understand what’s going on or… So community representatives I think are really important.* (Community Members Focus Group)

The need for support for people to participate in creative, cultural and community activities was emphasised by members of the CHWG. This may include transport infrastructure as well as childcare, respite for unpaid carers, and buddies to go to cultural activities and events with. There was also a suggestion around establishing supported voluntary and weekend roles in the cultural sector for people with additional needs.

This was also raised in the focus groups where it was suggested that a way of engaging communities was to create opportunities for community members themselves to be involved in and contribute to culture and health social prescribing and specifically outreach activities. Further suggestions included providing opportunities for students to build their skills (e.g. in design or advertising), volunteer outreach roles, training opportunities, and befriending or buddy schemes that support others to engage.

Another key example came from the local Recovery College which includes peer educators and volunteers in their service delivery.

*We’ve got about 19 staff members… at least six to seven of them are individuals who came as students to attend Recovery College and then became staff members… going back to that whole point about culture*,* we are trying to have this option where we are encouraging people to attend our courses and kind of having these real life examples where people are embracing the fact that they came as students and then became tutors or practitioners.* (Health Practitioners Focus Group)

Therefore, in conjunction with broader community level peer support positive role models were seen as ways to increase inclusion and engagement. However, members of the CHWG highlighted that culture and health provision across the region is not consistently available to everyone, it is dependent on location. Consistency was also referred to in terms of the social prescribing process only connecting people to activities for a set period. It was suggested that this “stop/start nature” can have a negative impact and does not meet the needs of people who would benefit from ongoing participation in activities. Similarly, the decline of existing longstanding activities was identified, with music opportunities in schools cited as an example. Such issues feed into wider concerns around the longevity of creative, cultural and community projects, long-term planning, and the sustainability of provision.

## Discussion

The specific purpose of this research was to support the development of approaches to the social prescribing of cultural opportunities in a district in the East of England, yet the findings are transferable to other areas where social prescribing occurs. We set out to establish what was happening locally in terms of culture and health provision, what already exists, what is working well, what is not working well, and where the gaps are to develop effective and appropriate structures and support. Structures and support are needed to ensure that social prescribers are aware of existing local provision, and to build the capacity of the creative sector to deliver arts, creativity and culture on prescription safely.

It was important to capture the views of members of the public as well as those of cultural providers, people working in the voluntary sector and those directly involved in social prescribing. A strength of this study was the inclusion of the wide range of stakeholders and the focus group methodology which enabled the views of all the participants to be captured and promote discussion through the sharing of experiences.

Patients are more likely to enrol in a social prescribing programme if it is presented to them in an acceptable way that matches their beliefs including whether they believe that it will benefit them [33]. Therefore, if members of the public are going to be willing to support and access social prescribing it is crucial that they understand what it is and what the potential benefits are of engaging with the process. For the most part the data suggest that the many of the those contributing to the focus groups had some idea of what social prescribing entailed but this was not the case universally, with some community members and even some healthcare professionals demonstrating a lack of understanding regarding the extent of what social prescribers did. However, generally amongst health and social care practitioners the term social prescribing is increasingly recognised, as the roles are introduced more widely around the UK. Other terms such as community referral have been used as an alternative name to social prescribing [34] and whilst the term social prescribing has been legitimised by its use in international and national policy documents and guidance, the word ‘prescription’ may be off putting for some people. This is because the risk of it being seen as medicalising social needs [35] and linked to the biomedical model of care with the associated perceived paternalistic approach of medical care [36, 37]. However, a contrasting view is that patients’ expectations regarding services and their preferences for health professionals may result in reluctance to seek support from the third sector or voluntary organisations [38]. Therefore, for some individuals who may have otherwise refused the use of the word ‘prescription’ may encourage them to take up the offer.

The language used to describe social prescribing has been described as diverse and confusing due to the lack of standardisation of names for social prescribing practitioners across different areas [39] but also on occasion within the same locality depending on where they are situated (e.g. link workers, care navigators, community connectors). In addition to the different labels assigned to similar roles in the UK, social prescribers can form part of primary care teams and be employed by the NHS but may also be employed by the voluntary sector and commissioned to work for the NHS [40]. Furthermore, different models of social prescribing are offered across different areas [33]. Such lack of consistency in use of terminology, and different models of service can confuse and create barriers to engagement and impair communication with healthcare professionals and the public. Effective communication and the use of appropriate language are important particularly for hard-to-reach groups who are already less likely to engage with social prescribing [37]. Social prescribing link workers therefore need awareness and sensitivity to a specific context, the local community and the characteristics of the participants referred to them [41]. It is recognised that there is a need for those initiating any form of community engagement to establish relationships to build trust and to be culturally competent, with knowledge of how information (and activities) will be received by different groups with translations and alternative communication formats available [42].

Difficulties with communication and understanding may exacerbate other access issues facing those in need. The research identified inequality of provision of cultural activities across different areas, lack of transport, the costs of attending activities and the time it takes to attend as possible barriers to accessing arts and cultural activities, even if activities are provided with no direct cost to the individual. This resonates with previous research, which also reported however that a modest cost to join a session could be a motivating factor [33]. The authors further suggested that to promote community engagement and inclusion the service must meet the needs of a local community, and it needs to be accessible. For some this means physical proximity to enable ease of travel to the sessions, for others it may also depend on the time of day the sessions are offered [33].

Social prescribing programmes are developed and delivered within the social and cultural context of a given community, where there will be different priorities and different expectations regarding where and to whom the programmes are targeted and what they are expected to achieve [43]. In the current research it was suggested that to increase inclusion the local community could be involved in the delivery of the service itself though a range of measures such as outreach and volunteering opportunities, support with language etc. Thomas et al. suggest that to promote engagement and inclusion that social prescribing programmes should be co-produced and co-designed with the local community to empower people and provide a sense of ownership in the programme [44]. However, whilst this maybe effective there will be those who are not represented and are excluded from engaging with co-production because of financial hardship, and competing priorities such as employment, and other structural barriers such as issues with language. Adams in 1989 (p.181) stated that “When participation is open to all it often becomes unequal” (cited in [45]) nevertheless lessons could be learnt from the wider literature regarding public and patient involvement in research. For example, research into how to be more inclusive and engage under-represented groups in patient and public involvement and engagement groups (PPIE) identified five key themes that need to be addressed; – to build trust, involvement from the beginning of a project, demonstrate impact, use clear and appropriate language, and imagine the life of the people you are trying to engage [46]. Equally, such strategies could also encourage engagement in social prescribing programmes.

As well as accessibility of different community resources and the activities that people can be referred to there is also a more fundamental issue and that is the availability of suitable resources and activities. This will depend on the local community infrastructure which as the participants in this current study described can be inconsistent across different areas. This means that in some areas there are few non-medical services or community assets for link workers to refer people to. Interventions that patients can be referred to as part of a social prescribing programme are commonly provided by third sector organisations such as charities, community groups, and volunteers [20]. However, the third sector in the UK has been affected by the lack of government support and there is therefore a need for funding for voluntary and community sector organisations if they are to be able to act as delivery partners [47]. Where funding is provided for community assets to support social prescribing initiatives these may be time limited meaning that there may be insufficient time for patients with longstanding or complex conditions to benefit from the activity or intervention they are referred to [48]. The National Academy for Social Prescribing has recently highlighted the need for an England- wide mechanism for social investment to be adopted to increase community capacity to deliver interventions. This would have the potential to widen the reach and range of services which offer interventions and enable the collaboration between statutory and community providers [49].

## Limitations

Although the research was conducted in one region in the UK, the issues around terminology, inclusion, and community engagement have been identified in the wider healthcare literature indicating that the findings would apply elsewhere. The research is limited by the fact that those who consented to take part may have been those who already knew about social prescribing and had a vested interested in offering their perspectives on local cultural provision or lack of it. Secondly, as raised in the discussion section it can be difficult for some people to participate in research because of accessibility issues including language, time commitments, and the financial implications of taking part. For further research the suggestions made regarding how to increase representation from all sectors of the community should be considered.

## Conclusions

This research indicates the need to develop a clear understanding of what is meant by ‘culture and health’. Using examples of cultural opportunities and their potential to promote health and wellbeing could be instrumental in developing an understanding of how culture and cultural opportunities can link and contribute to social prescribing (or otherwise). Whilst the focus of this research was on culture and health and the opportunities for cultural engagement through social prescribing the issues around terminology and inclusion are relevant to all models of social prescribing. More broadly, the research adds to the existing international evidence base demonstrating the importance of involving local stakeholders including community members in the development of services to optimise provision and take up by those in most need.

## Data Availability

The datasets generated and/or analysed during the current study are available from the corresponding author on reasonable request.
